# Changes in thalamic metabolites correlate with severity of spinal cord injury based on diffusion tensor imaging in patients with cervical spondylotic myelopathy

**DOI:** 10.3389/fneur.2026.1791063

**Published:** 2026-04-13

**Authors:** Xiaodan Mu, Yujin Zhang, Yuan Yao, Baogen Zhao, Wei Yan, Li Zhang

**Affiliations:** 1 Department of Radiology, Peking University International Hospital, Beijing, China; 2 Department of Radiology and Nuclear Medicine, The First Hospital of HeBei Medical University, Shijiazhuang, China; 3 Department of Ultrasound, Shijiazhuang People’s Hospital, Shijiazhuang, China; 4 Health Care Center, The First Hospital of Hebei Medical University, Shijiazhuang, China

**Keywords:** cervical spondylotic myelopathy, correlation, diffusion tensor imaging, dorsal thalamus, magnetic resonance spectroscopy

## Abstract

**Purpose:**

To investigate the changes in thalamic metabolites in patients with cervical spondylotic myelopathy (CSM) using magnetic resonance spectroscopy (MRS) and their correlation with the severity of spinal cord injury based on diffusion tensor imaging (DTI).

**Materials and methods:**

A total of 93 CSM patients and 67 healthy controls (HCs) were enrolled in this prospective study from December 2023 to September 2024. All participants underwent routine magnetic resonance imaging (MRI) of the cervical spine, MRS of the bilateral thalamus, and DTI of cervical spine. Parameters of DTI (FA, ADC) and MRS (NAA/Cr, Cho/Cr, MI/Cr, and Glx/Cr) were compared between the CSM patients and HCs. Correlation analyses were performed among clinical characteristics, MRI features of cervical spine, and DTI and MRS parameters in CSM patients. Multivariate linear regression equations for thalamic metabolites were established based on these features.

**Results:**

FA values in the CSM group were significantly lower than those in the HCs (*p* = 0.005, *t* = 2.874). The CSM group exhibited significantly reduced NAA/Cr (*p* < 0.001, *Z* = −5.922), Cho/Cr (*p* < 0.001, *Z* = −6.857), and MI/Cr ratios (*p* < 0.001, *Z* = −5.922) compared to the HCs. In the CSM group, NAA/Cr (*r* = 0.444, *p* < 0.001), Cho/Cr (*r* = 0.308, *p* = 0.003), and MI/Cr (*r* = −0.489, *p* < 0.001) correlated with FA values. NAA/Cr also correlated with symptom duration (*r* = −0.365, *p* < 0.001). The multivariate linear regression equations for thalamic metabolites were as follows: NAA/Cr = 0.833 + 1.520*FA-0.007*duration; Cho/Cr = 0.209 + 0.774*FA; MI/Cr = 1.566–1.722*FA + 0.008*mJOA scores; Glx/Cr = 0.942 + 0.009*duration.

**Conclusion:**

CSM patients exhibit metabolic alterations in the dorsal thalamus, which are linearly correlated with the severity of spinal cord injury.

## Introduction

Cervical spondylotic myelopathy (CSM) is characterized by cervical intervertebral disc degeneration accompanied by secondary pathological changes in adjacent structures, such as bone hyperplasia, joint hypertrophy, and ligament calcification (1). These changes lead to clinical manifestations including neck pain, restricted mobility, limb numbness, muscle weakness, and dizziness. In advanced stages, potential paralysis may occur, significantly impacting quality of life (2, 3). Early diagnosis and assessment are therefore crucial for improving prognosis.

Research indicates that CSM arises from chronic cervical spinal cord compression-induced nerve fiber damage, particularly in the lateral corticospinal tract (4). Previous studies have predominantly focused on spinal cord lesions (5–7). However, emerging evidence shows inconsistencies between clinical symptoms and the degree of spinal cord injury in some patients. Notably, postoperative patients often demonstrate early motor improvement, while sensory recovery lags or remains incomplete (8–10). This suggests that CSM is associated with not only direct spinal cord damage but also remote alterations in higher brain centers. Such alterations may reflect adaptive or maladaptive responses to chronic cord compression, potentially representing the neural substrate for sensory-motor function recovery after surgical decompression (11). The thalamus, rich in neurons, functions as a critical sensory relay station and movement regulator. Particularly sensitive to ischemia and hypoxia, thalamic neurons are vulnerable to structural and functional impairments (12, 13). Recent studies have identified functional and structural alterations in the dorsal thalamus of CSM patients, with metabolite levels serving as important biomarkers of thalamic injury and potential adaptive changes (14, 15). However, existing research has not yet elucidated the correlation between these metabolic changes in the thalamus and the severity of spinal cord injury.

Although conventional magnetic resonance imaging (MRI) can clearly reveal morphological changes and signal alterations in the spinal cord, it fails to reflect microstructural variations or to enable quantitative evaluation, thus exhibiting limited sensitivity for spinal cord injury (16). Recent advancements in medical imaging technology have provided new opportunities for research on CSM. Diffusion tensor imaging (DTI) quantifies water molecule diffusion patterns in biological tissues to detect subtle structural changes in living neural fibers, utilizing multiple parametric values to quantitatively assess spinal cord injury and to reveal microscopic information regarding neural fiber connectivity and pathophysiological alterations within the spinal cord (17). Fractional anisotropy (FA), the most commonly used DTI metric, reflects the degree of this directional preference and serves as a surrogate marker of white matter tract integrity. FA values are reduced in pathological conditions affecting white matter, such as demyelination, axonal degeneration, or ischemia (18). Magnetic resonance spectroscopy (MRS) currently stands as the only non-invasive methodology capable of investigating metabolic processes, biochemical changes, and metabolite quantification in living tissues, enabling sensitive detection of cerebral metabolic alterations prior to structural abnormalities (19). Creatine is involved in cellular energy metabolism and is generally considered more stable than other metabolites under pathological conditions.

This study therefore aims to investigate metabolic changes in the dorsal thalamus of CSM patients using MRS and to analyze their correlation with the severity of spinal cord injury evaluated by DTI.

## Materials and methods

### Participants

This study was performed in compliance with relevant laws and institutional guidelines and was approved by the Medical Ethics Committee (S0025320221107). A total of 93 CSM patients treated between December 2023 and September 2024 were enrolled in the patient group, along with 67 age- and sex-matched healthy volunteers selected as the healthy controls (HCs). Inclusion criteria were as follows: ① Typical clinical symptoms and signs of CSM; ② No contraindications to MRI examination. Exclusion criteria were as follows: ① Lactating or pregnant women; ② a history of cervical spine surgery; ③ Spinal cord tumors, trauma, inflammatory diseases, or demyelinating disorders; ④ Central nervous system disorders or psychiatric diseases. Spinal cord function was assessed using the modified Japanese Orthopaedic Association (mJOA) score, with a total possible score of 18 points. The privacy rights of human subjects were observed, and written informed consent was obtained from all participants or their legal guardians.

### MRI data acquisition

Routine MRI of the cervical spine, ^1^H-MRS of the bilateral thalamic, and DTI of the cervical spine were performed using a Philips Ingenia-cx 3.0 T MRI system equipped with a 20-channel head–neck combined coil. Scanning Parameters: sagittal T1-weighted imaging (T1WI): TR 590 ms, TE 7.5 ms, FOV 160 mm × 229 mm. Sagittal T2-weighted imaging (T2WI): TR 2500 ms, TE 25 ms, FOV 161 mm × 232 mm. Short Tau Inversion Recovery (STIR): TR 2500 ms, TE 60 ms, FOV 160 mm × 228 mm. For DTI, automatic shimming and fat suppression techniques were applied. Sagittal imaging was performed using a single shot spin echo sequence with the following parameters: TR 5000 ms, TE 95 ms, FOV 240 mm × 240 mm, slice thickness 3 mm. Diffusion-sensitizing gradients were applied in 10 directions with b-values of 0 and 1,000 s/mm^2^. For MRS, the largest thalamic cross-section was selected for voxel placement. Single-voxel spectroscopy was acquired using the Point-Resolved Spectroscopy (PRESS) sequence with the following parameters: TR 2000 ms, TE 30 ms, 128 signal averages, and a voxel size of 13 mm × 13 mm × 16 mm. Spectral quality control was rigorously assessed using the following criteria: (1) signal-to-noise ratio (SNR) > 10 for all metabolites of interest; (2) full width at half maximum (FWHM) of the water peak < 10 Hz; (3) Cramér-Rao lower bounds (CRLB) < 20% for all metabolites. Spectra failing to meet any of these criteria were excluded from analysis. Although we did not perform voxel tissue composition correction or cerebrospinal fluid correction for the MRS data, the thalamus is a relatively homogeneous gray matter structure, and careful voxel placement minimized CSF contamination (20). All post-processing was performed on workstation (IntelliSpace Portal, Philips). Efforts were made to minimize cerebrospinal fluid contamination during voxel positioning. Two radiologists with differing levels of neuroimaging diagnostic experience (3 years and 7 years) conducted double-blind evaluations, and discrepancies were resolved through consensus discussion.

### DTI acquisition and analysis

**Figure 1:**
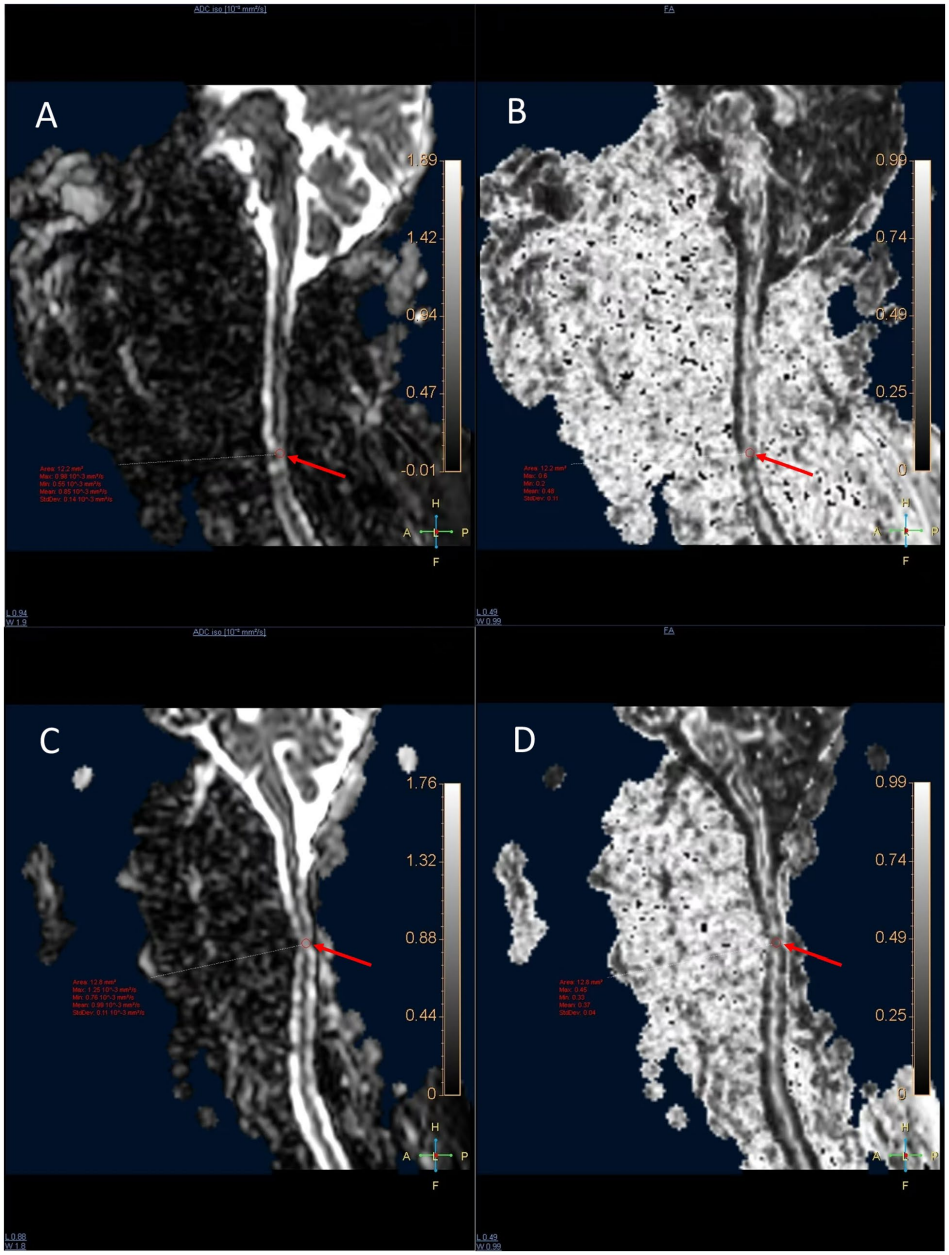
Sagittal DTI of the cervical spine in two patients with CSM. **(A, B)** A 63-year-old male patient: **(A)** FA map shows reduced FA signal at the C5–C6 level, **(B)** ADC map shows elevated ADC signal at the same level. **(C, D)** A 48-year-old male patient: **(C)** FA map shows reduced FA signal at the C3–C4 level, **(D)** ADC map shows elevated ADC signal at the C3–C4 level. In CSM patients, ROIs were placed at the level of maximal compression or T2 hyperintensity to capture the most severe microstructural damage.

Following distortion correction, FA and ADC maps were generated, and FA and ADC values were measured in the sagittal plane. In CSM patients, regions of interest (ROIs) were placed at the level of maximal cord compression or at the site of T2 hyperintensity, as this location represents the epicenter of white matter injury where microstructural damage is most pronounced (21). In healthy controls, ROIs were placed at the C5-C6 intervertebral disc level, the most common site of spondylotic change (22), based on evidence that FA values do not differ significantly across cervical segments in normal individuals (23). All ROIs covered an area of 10–15 mm^2^. Measurements were repeated three times and averaged, as illustrated in Figure 1.

### MR spectroscopy and analysis

**Figure 2:**
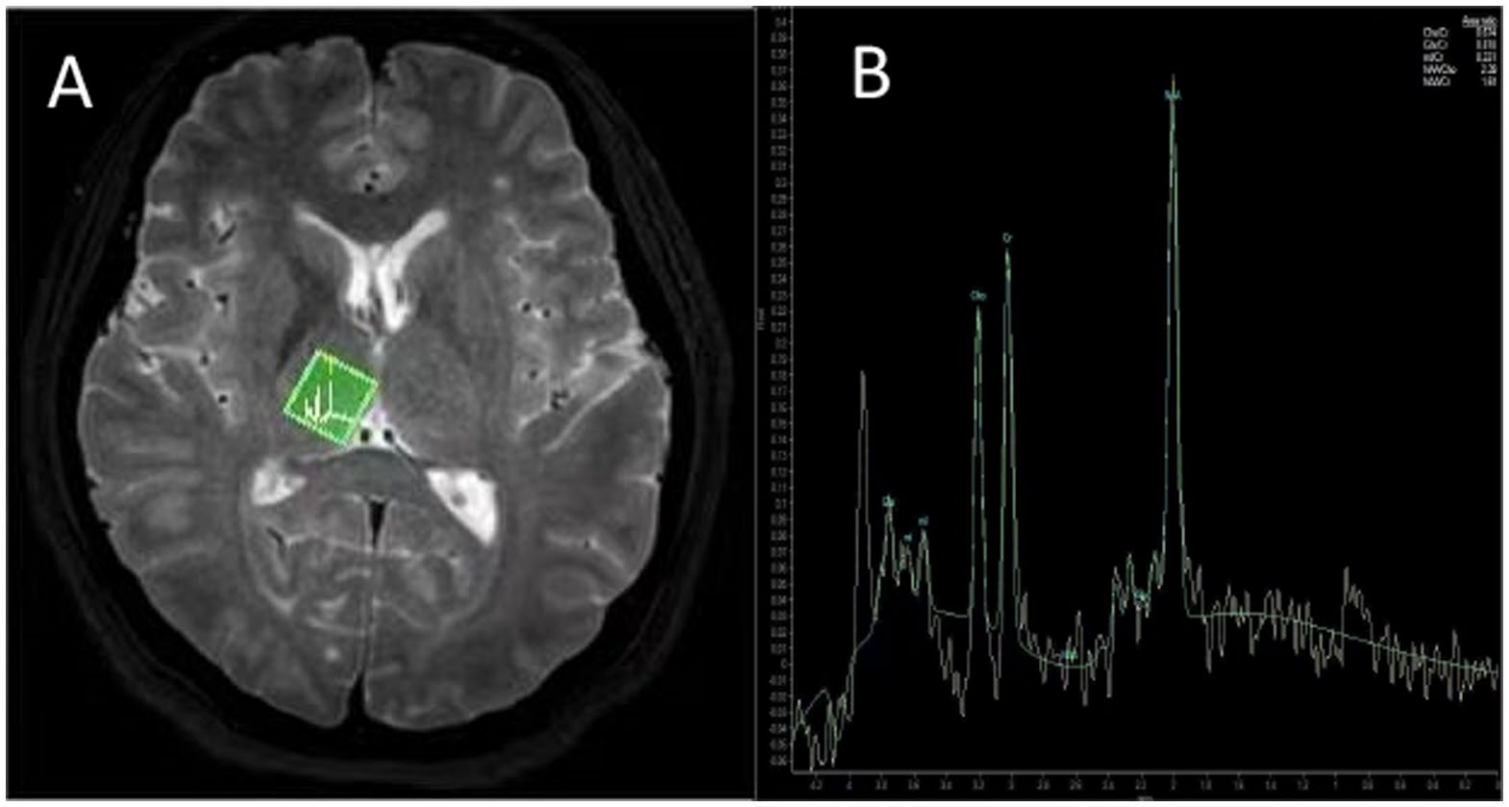
^1^H-MRS of the right thalamus in a 57-year-old male patient with CSM **(A)**. Axial T1-weighted image showing the voxel was carefully positioned at the largest thalamic cross-section, avoiding the ventricular margins to minimize cerebrospinal fluid contamination **(B)**. Corresponding MR spectrum with metabolite peaks labeled, the metabolite ratios (NAA/Cr, Cho/Cr, MI/Cr, Glx/Cr) were calculated from the fitted peak areas.

Using the Spectroview software package, ROIs were carefully positioned at the largest thalamic cross-section, covering an area of 10–15 mm^2^, with bilateral measurements averaged. The analyzed metabolites included: N-acetyl aspartate (NAA), Choline (Cho), Creatine (Cr), Myo-inositol (MI), Glutamate (Glx). Metabolite ratios were recorded as NAA/Cr, Cho/Cr, MI/Cr, and Glx/Cr, as illustrated in Figure 2.

### Statistical analysis

Data were analyzed using SPSS 21.0. Normally distributed data were expressed as mean ± standard deviation (SD) and compared using independent samples *t*-test. Non-normally distributed data were expressed as median (25, 75%) [M (25, 75%)], compared using Mann–Whitney *U* test. Correlation analysis for non-normal distributions used Spearman’s rank correlation coefficient. Multiple linear regression analysis was conducted to identify factors independently associated with each thalamic metabolite ratio (NAA/Cr, Cho/Cr, mI/Cr, Glx/Cr). Candidate independent variables included DTI parameters and clinical indicators. Multiple linear regression analysis was performed using a stepwise selection method (entry criterion *p* < 0.05, removal criterion *p* > 0.10). This approach was chosen to identify the most parsimonious set of predictors independently associated with each thalamic metabolite ratio. To account for multiple comparisons across the parameters, a Bonferroni correction was applied, with the significance threshold set at *α* = 0.05/4 = 0.0125. The statistical significance level was defined as *α* = 0.05, *p* < 0.05. Inter-rater reliability for all DTI parameters (FA and ADC) and MRS metabolite ratios was assessed using intraclass correlation coefficients (ICC). ICC values were interpreted as follows: < 0.50 indicated poor reliability, 0.50–0.75 moderate reliability, 0.75–0.90 good reliability, and > 0.90 excellent reliability (24).

## Results

### Participants characteristics

The study ultimately enrolled 93 patients and 67 HCs. No significant differences were observed between CSM patients and HCs in terms of gender (*χ*^2^ = 3.063, *p* = 0.087), handedness (*χ*^2^ = 0.072, *p* = 0.788), or age (*Z* = −0.882, *p* = 0.377). Detailed clinical characteristics of the participants are presented in Table 1.

**Table 1:** Characteristics of CSM Patients and HCs.

Clinical characteristic	CSM (*n* = 93)	HCs (*n* = 67)	*p*
Age (y)^a^	52.67 ± 11	53.13 ± 13	0.377
Genders (%)^a^			0.087
Male	69 (74.2)	41 (61.2)	
Female	24 (25.8)	26 (38.8)	
Duration (month)	9.4 ± 4	–	
T2WI hyperintensity (%)	17 (18.3)	–	
Dominant hand (%)^a^			0.788
Right-handed	78 (83.9)	56 (83.6)	
Left-handed	8 (8.6)	5 (7.5)	
Ambidextrous	7 (7.5)	6 (8.9)	
mJOA score	11.60 ± 3.05	18.00 ± 0.00	

CSM, Cervical Spondylotic Myelopathy; HCs, healthy controls. ^a^Normally distributed continuous variables (mean ± SD) were statistically analyzed by Student’s *t*-test.

### Inter-rater reliability

Inter-rater reliability was good to excellent for all DTI and MRS measurements. The ICC for FA was 0.91 (95% CI: 0.87–0.94), for ADC, 0.88 (95% CI: 0.83–0.92), for NAA/Cr, 0.93 (95% CI: 0.89–0.95), for Cho/Cr, 0.90 (95% CI: 0.86–0.93), for MI/Cr, 0.89 (95% CI, 0.84–0.92), and for Glx/Cr, 0.85 (95% CI, 0.80–0.89). These values indicate a high degree of agreement between the two radiologists.

### MRS data quality

Detailed MRS quality control metrics, including SNR, FWHM, and CRLB values for all metabolites stratified by group, are provided in Supplementary Table S1. All parameters met the predefined quality criteria, and no significant differences were observed between CSM patients and HCs.

### DTI and MRS parameters

**Figure 3:**
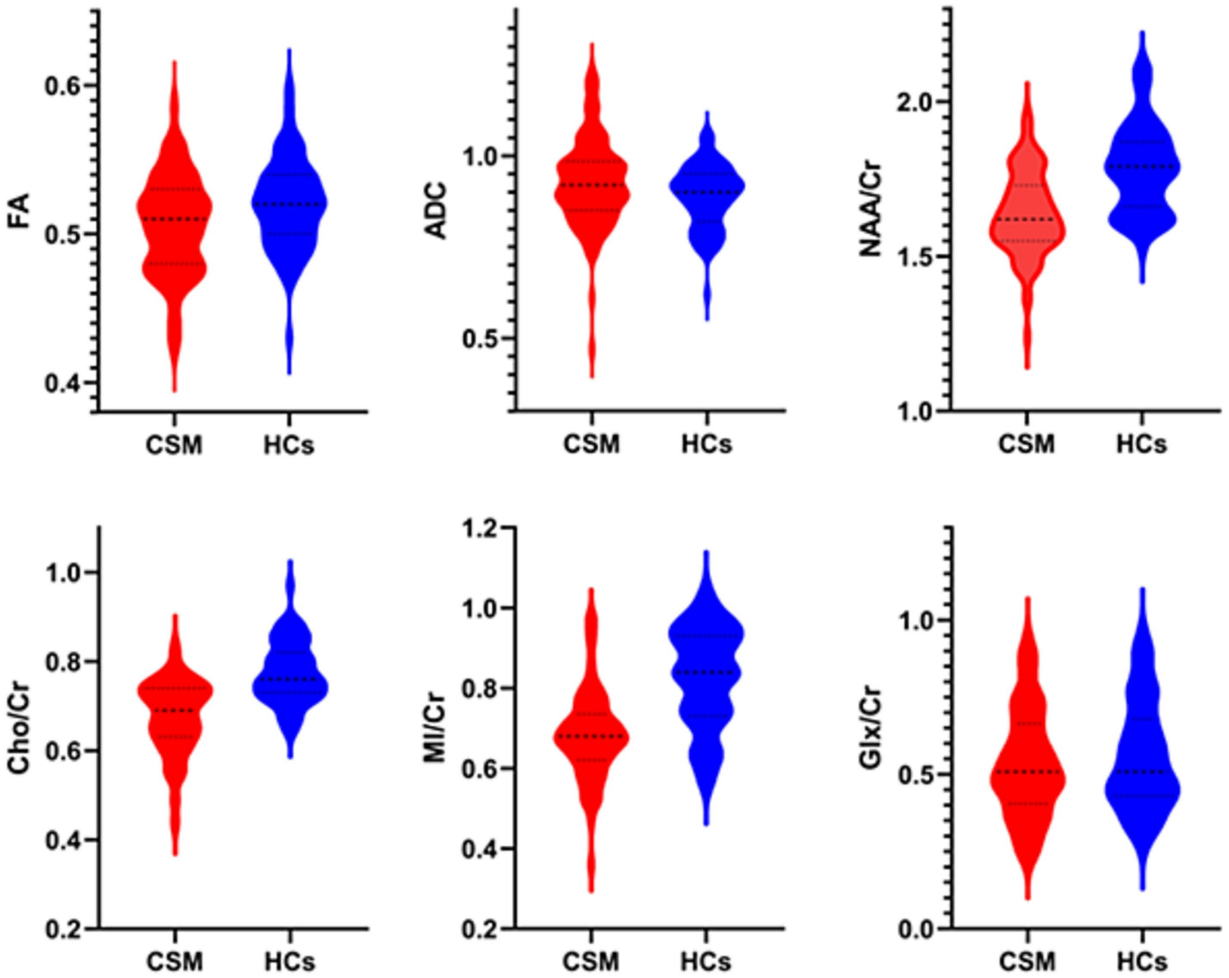
Violin plots comparing DTI parameters and MRS metabolite ratios between patients with CSM and HCs, the width of the shaded area represents the probability density of the data at different values.

After applying Bonferroni correction for multiple comparisons, partial parameters remained statistically significant. FA values were significantly lower in the patients group compared to HCs (*p* = 0.005, *t* = 2.874). No significant difference was observed in ADC values (*p* = 0.050, *Z* = −1.959). The patient group exhibited significantly reduced metabolite ratios compared to HCs: NAA/Cr (*p* < 0.001, *Z* = −5.922), Cho/Cr (*p* < 0.001, *Z* = −6.857), and MI/Cr (*p* < 0.001, *Z* = −5.922). No significant difference was found in Glx/Cr (*p* > 0.05, *Z* = −0.68) (Figure 3, Table 2).

**Table 2:** DTI and MRS results of the CSM and HCs.

Parameter	CSM (*n* = 93)	HCs (*n* = 67)	*p* value	*t*/*Z*
FA	0.505 ± 0.034	0.520 ± 0.030	0.005	2.874^a^
ADC (×10^−3^ mm^2^/s)	0.916 (0.851, 0.985)	0.904 (0.821, 0.946)	0.050	−1.959^b^
NAA/Cr	1.615 (1.550, 1.730)	1.785 (1.655, 1.865)	0.000	−5.922^b^
Cho/Cr	0.691 (0.626, 0.737)	0.760 (0.727, 0.822)	0.000	−6.857^b^
MI/Cr	0.680 (0.621, 0.737)	0.839 (0.730, 0.933)	0.000	−6.573^b^
Glx/Cr	0.508 (0.402, 0.663)	0.508 (0.425, 0.675)	0.497	−0.680^b^

DTI, diffusion tensor imaging; MRS, magnetic resonance spectroscopy; HCs, healthy controls. ^a^Normally distributed continuous variables (mean ± SD) were statistically analyzed by Student’s *t*-test. ^b^Nonnormally distributed continuous variables (median [IQR]) were statistically analyzed by the Mann Whitney *U* test.

### Correlation and multiple linear regression analyses

**Figure 4:**
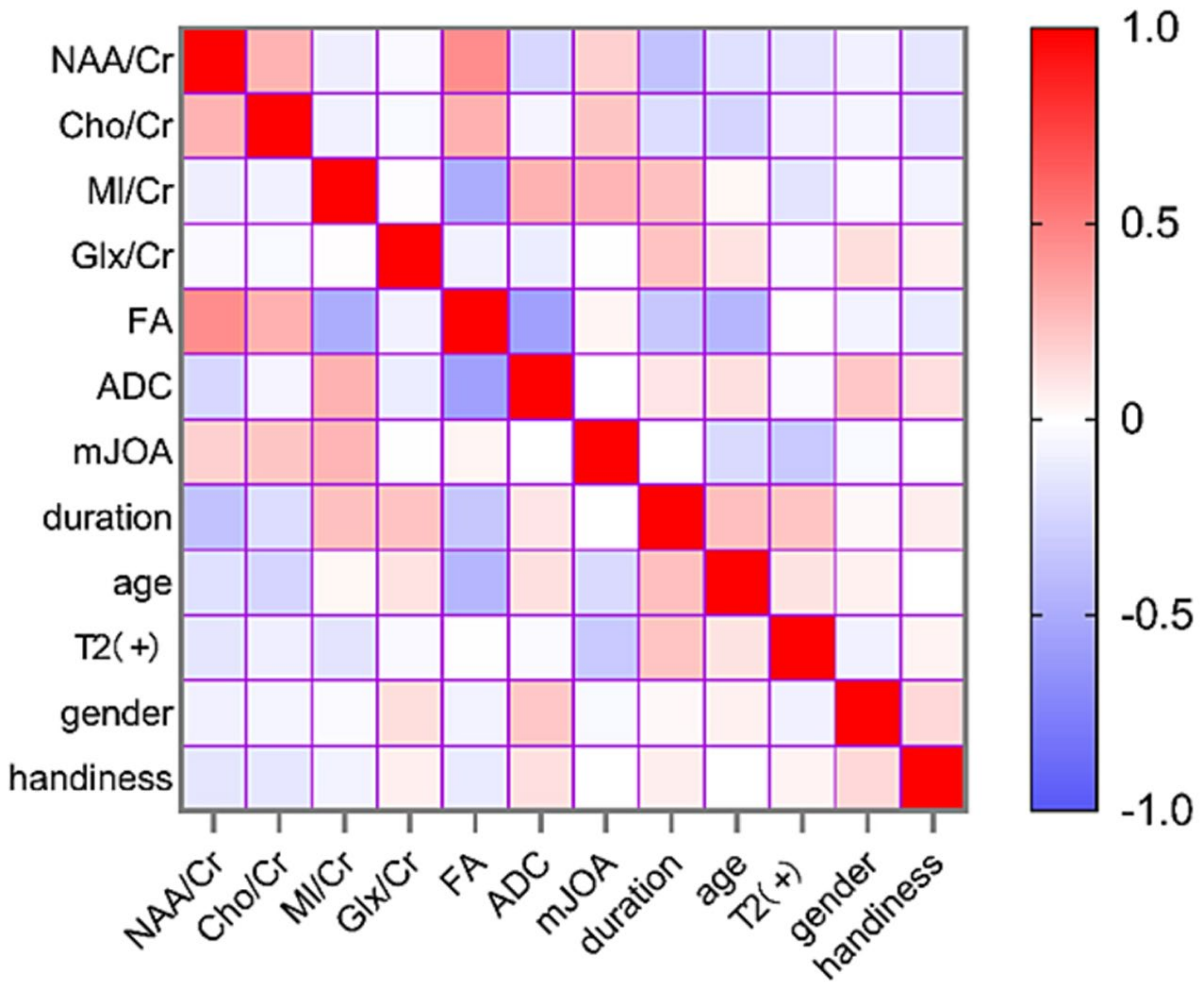
Heatmap showing Spearman’s rank correlation coefficients (*r*) between thalamic metabolite ratios, DTI parameters, and clinical indicators in patients with CSM. The color scale ranges from dark blue (strong negative correlation, *r* = −1.0) through white (no correlation, *r* = 0) to dark red (strong positive correlation, *r* = +1.0).

**Figure 5:**
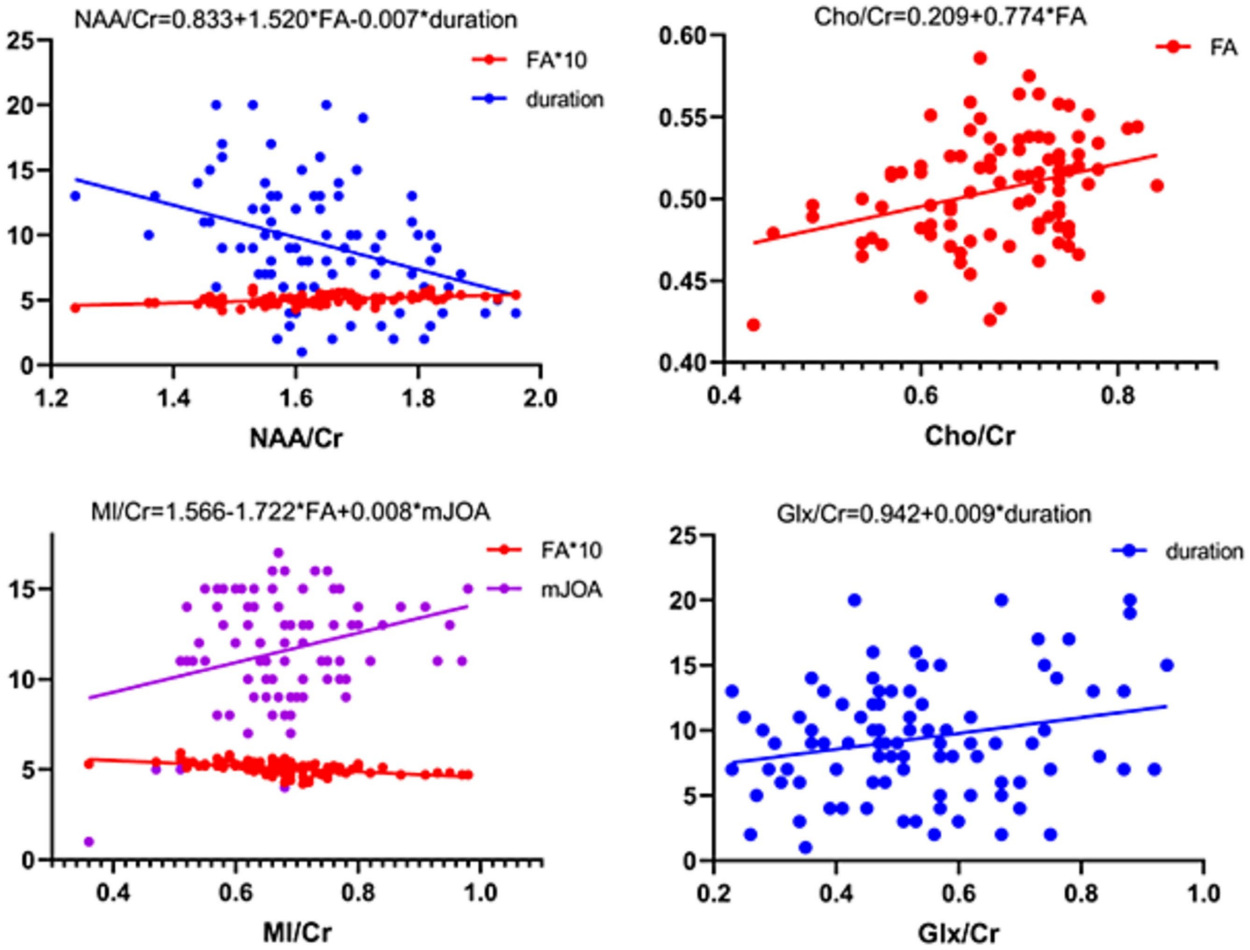
Multivariate linear regression models for thalamic metabolite ratios in patients with CSM. Each model was derived using stepwise selection from candidate predictors including DTI parameters (FA, ADC) and clinical variables (sex, age, handedness, mJOA score, presence of T2WI hyperintensity, disease duration). Equations represent the final parsimonious models.

All VIF values are <2, well below the recommended threshold of 5, indicating no problematic multicollinearity among predictors. Both histograms and P–P plots indicated that the standardized residuals approximated a normal distribution. Scatterplots of standardized residuals versus standardized predicted values showed a random distribution, confirming constant variance. The Durbin–Watson statistic was also calculated, and all models fell within the acceptable range of 1.5 to 2.5, indicating no significant autocorrelation. No observations had an absolute standardized residual >3, confirming the absence of influential outliers. In the Patient Group, NAA/Cr (*r* = 0.444, *p* < 0.001), Cho/Cr (*r* = 0.308, *p* = 0.003), and MI/Cr (*r* = −0.489, *p* < 0.001) correlated with FA values. NAA/Cr correlated inversely with symptom duration (*r* = −0.365, *p* < 0.001) (Figure 4). Metabolite ratios (dependent variables) were modeled against FA, ADC, and clinical indicators (sex, age, handedness, mJOA score, spinal cord T2WI hyperintensity, disease duration): NAA/Cr = 0.833 + 1.520 × FA − 0.007 × duration, Cho/Cr = 0.209 + 0.774 × FA, MI/Cr = 1.566–1.722 × FA + 0.008 × mJOA scores, Glx/Cr = 0.942 + 0.009 × duration (Figure 5, Table 3).

**Table 3:** Multiple linear regression analyses results.

MRS ratio	Independent variable	*b* value	*t*	*p* value	*R*^2^	Adjusted *R*^2^
NAA/Cr	FA	1.520	3.008	0.003	0.300	0.234
	duration	−0.007	−2.451	0.016		
Cho/Cr	FA	0.774	2.296	0.024	0.191	0.113
MI/Cr	FA	−1.722	−4.526	0.000	0.395	0.337
	mJOA score	0.008	2.486	0.015		
Glx/Cr	duration	0.009	2.083	0.040	0.131	0.049

MRS, magnetic resonance spectroscopy; mJOA, modified Japanese Orthopedic Association score.

## Discussion

This study firstly demonstrated that FA values and thalamic metabolites can differentiate CSM patients from HCs. Furthermore, correlations were identified between NAA/Cr, Cho/Cr, and MI/Cr ratios with FA values.

Following cervical spinal cord compression, pathological changes initially manifest as atrophy and degeneration of gray matter neurons, followed by white matter demyelination and axonal degeneration (25). DTI quantitatively reflects microstructural pathological alterations in spinal cord nerve fibers (26): FA values reflect the density of white matter fibers and myelin sheath integrity, while ADC values indicate the overall diffusion intensity of water molecules. Under chronic compression, ischemia and hypoxia induce partial disruption of cell membranes and myelin sheaths occurs, accompanied by axonal tearing (27). This reduces the anisotropic diffusion of water molecules along fiber tracts, resulting in decreased FA values (28). Progressive compression exacerbates hypoperfusion, increasing membrane permeability and fiber loss. ADC values—while showing a trend toward elevation—did not differ significantly between groups (*p* = 0.05). This may reflect that the degree of cellular necrosis and extracellular edema was not sufficiently enough to produce measurable diffusivity changes. We refrain from detailed physiological interpretation of the ADC finding. Thus, FA anisotropy appears to be a more sensitive biomarker than diffusivity for detecting early microstructural damage in CSM.

The thalamus is a deep gray matter nucleus in the brain, containing abundant neurons that regulate somatic movement and play a crucial role in sensory processing and signal modulation (29). Early stages of ischemia and hypoxia may cause structural and functional damage to thalamic neurons (19). Alterations in metabolites within organisms typically precede structural tissue changes, and MRS can quantitatively reflect molecular pathological changes. NAA reflects neuronal structure and function, with decreased levels indicating neuronal loss or damage (30). The negative MI/Cr-FA association indicates that more severe spinal cord microstructural damage (lower FA) is associated with higher thalamic MI/Cr, suggesting that MI serves as a marker of glial proliferation or activation (31). In CSM, we propose that chronic cord compression induces retrograde axonal injury, triggering glial activation in thalamic projection zones and leading to elevated MI detectable by MRS. Study (32) has reported segmental neuronal loss, oligodendrocyte depletion, and astrocyte reduction in rats with cervical spinal cord compression. Therefore, changes in MRS parameters in the thalamus may be attributed to decreased blood perfusion caused by thalamic injury, leading to tissue ischemia and hypoxia, abnormal neuronal metabolism, and subsequently thalamic neuronal damage and functional decline (33). As neurons and oligodendrocytes progressively degenerate, the integrity of white matter tracts becomes compromised, accompanied by corresponding decreases in metabolites.

This study demonstrates a correlation between thalamic metabolites and FA values, with FA values showing significant associations with NAA/Cr, Cho/Cr, and MI/Cr in linear regression analyses. Consequently, the structural and functional disturbances in the cervical spinal cord and thalamus observed in this study may be attributed to retrograde axonal injury caused by chronic spinal cord compression, leading to thalamic neuronal atrophy, degeneration, and necrosis. At the molecular level, Wu et al. demonstrated that chemokines, inflammatory factors, and damage-associated molecular patterns produced at the spinal injury site are retrogradely transmitted through the corticospinal tract to specific brain regions, where they trigger pathologic changes. This establishes a biologically plausible pathway for remote effects following spinal cord damage (34). Kang et al. (35) found that DTI metrics in specific thalamic nuclei differed significantly based on injury severity in pediatric SCI patients. This might contribute to gray matter loss in the thalamus and reduce thalamic sensorimotor connectivity (33, 36). Importantly, the hypothesis that CSM induces neuroplastic changes in the brain requires direct testing with longitudinal designs; our research group is actively pursuing this question.

Our study has several limitations. First, FA values at the fixed C5–C6 level in controls introduces potential systematic bias with those at variable compression levels in patients. This could lead to overestimation or underestimation of true group differences. However, prior evidence suggests minimal segmental FA variation in healthy individuals (23), which mitigates this concern. Any measurement error introduced by inconsistent ROI placement potentially weaken the observed correlations between FA and thalamic metabolites. Given that we observed consistent correlations across multiple metabolites, the impact of such bias on our conclusions is likely limited. Nevertheless, to definitively address this methodological limitation, future studies should employ multi-level ROI sampling or whole-cord tractography, which would eliminate group-specific ROI placement and enable more robust correlation analyses. Second, a methodological limitation is the lack of gray and white matter segmentation in our spinal cord DTI analysis. Although whole-cord FA is well-validated as a measure of white matter integrity and our primary research question focused on overall spinal cord damage, segmentation would enable tract-specific analysis. Third, we acknowledge that stepwise regression, while useful for exploratory analyses, has inherent limitations including potential overfitting and dependence on the specific entry/removal thresholds. The final models should therefore be interpreted as hypothesis-generating and require validation in independent cohorts. Fourth, we used metabolite ratios relative to Cr rather than absolute quantification. This approach is widely employed in clinical MRS studies and has been successfully applied in CSM investigations^1,2^. Nevertheless, future studies (37) using absolute quantification with water referencing and tissue-segmentation correction would provide additional confirmation of our findings. Fifth, although we applied Bonferroni correction to control the family-wise error rate across the parameters, this approach is conservative and may increase the risk of Type II errors (false negatives), particularly for comparisons with modest effect sizes. The ADC comparison fell below the adjusted threshold. This borderline result may represent either a true difference undetected due to insufficient power (Type II error) or a chance finding, and should be considered when interpreting near-significant findings. The risk of false positives inherent in multiple testing is a recognized limitation of studies with multiple outcome measures. In conclusion, this study demonstrates that CSM patients exhibit reduced thalamic NAA/Cr, Cho/Cr, and MI/Cr ratios, and that these metabolite levels are correlated with FA values. Given the cross-sectional design, these results represent associations rather than causal relationships. These findings indicate that spinal cord microstructural damage and thalamic neurochemical changes are interrelated in CSM, suggesting that spinal cord FA may serve as a predictive biomarker for thalamic metabolic alterations.

## Data Availability

The raw data supporting the conclusions of this article will be made available by the authors, without undue reservation.
