# Supplementary material for: Changes in thalamic metabolites correlate with severity of spinal cord injury based on diffusion tensor imaging in patients with cervical spondylotic myelopathy
Source: Front Neurol. 2026 Apr 13;17:1791063. doi: 10.3389/fneur.2026.1791063 (PMC13110980; doi:10.3389/fneur.2026.1791063)
Supplement: Supplementary file 1 [file Table_1.DOCX]

Table S1 MRS Quality Control Metrics

| Parameter | CSM (n=93) | HCs (n=67) | Overall (N=160) |
| --- | --- | --- | --- |
| SNR |  |  |  |
| NAA | 18.4 ± 3.1 (12–25) | 18.6 ± 3.3 (13–26) | 18.5 ± 3.2 (12–26) |
| Cr | 16.2 ± 2.8 (10–22) | 16.5 ± 3.0 (11–23) | 16.3 ± 2.9 (10–23) |
| Cho | 14.8 ± 2.5 (9–20) | 15.1 ± 2.7 (10–21) | 14.9 ± 2.6 (9–21) |
| MI | 11.3 ± 2.2 (7–16) | 11.6 ± 2.4 (8–17) | 11.4 ± 2.3 (7–17) |
| Glx | 9.8 ± 1.9 (6–14) | 10.1 ± 2.1 (7–15) | 9.9 ± 2.0 (6–15) |
| FWHM (Hz) | 5.3 ± 0.8 (4–7) | 5.1 ± 0.7 (4–7) | 5.2 ± 0.8 (4–7) |
| CRLB (%) |  |  |  |
| NAA | 5.2 ± 1.1 (3–8) | 4.9 ± 1.0 (3–7) | 5.1 ± 1.1 (3–8) |
| Cr | 6.1 ± 1.3 (4–9) | 5.8 ± 1.2 (4–8) | 6.0 ± 1.3 (4–9) |
| Cho | 7.2 ± 1.5 (5–11) | 6.9 ± 1.4 (5–10) | 7.1 ± 1.5 (5–11) |
| MI | 12.3 ± 2.4 (8–18) | 11.8 ± 2.2 (8–17) | 12.1 ± 2.3 (8–18) |
| Glx | 15.1 ± 2.8 (10–20) | 14.7 ± 2.6 (10–19) | 14.9 ± 2.7 (10–20) |

Data presented as mean ± SD. No significant differences were observed between groups for any parameter (all *P* > 0.05). Abbreviations: SNR, signal-to-noise ratio; FWHM, full width at half maximum; CRLB, Cramér-Rao lower bounds.
